# Viruses Binding to Host Receptors Interacts with Autophagy

**DOI:** 10.3390/ijms24043423

**Published:** 2023-02-08

**Authors:** Jinsung Yang

**Affiliations:** Department of Biochemistry and Convergence Medical Science, Institute of Health Sciences, College of Medicine, Gyeongsang National University, Jinju 52727, Republic of Korea; jyang@gnu.ac.kr

**Keywords:** virus, autophagy, entry, receptor, infection, lysosome, attachment factor, cell surface molecules

## Abstract

Viruses must cross the plasma membrane to infect cells, making them eager to overcome this barrier in order to replicate in hosts. They bind to cell surface receptors as the first step of initiating entry. Viruses can use several surface molecules that allow them to evade defense mechanisms. Various mechanisms are stimulated to defend against viruses upon their entry into cells. Autophagy, one of the defense systems, degrades cellular components to maintain homeostasis. The presence of viruses in the cytosol regulates autophagy; however, the mechanisms by which viral binding to receptors regulates autophagy have not yet been fully established. This review discusses recent findings on autophagy induced by interactions between viruses and receptors. It provides novel perspectives on the mechanism of autophagy as regulated by viruses.

## 1. Introduction

Viruses use hosts’ intracellular functions to multiply, so they must cross the cell membrane [1]. A viral infection is a complex process requiring a coordinated series of cell surface molecules for viruses to enter cells [2]. Viral entry begins with binding to the surface via adhesion factors such as cell surface glycans [3]. Afterward, viruses search for specific receptors and initiate entry [4,5]. This process often requires conformational changes in viral proteins and receptors, making the procedure highly organized [2]. The mechanisms for viral internalization are diverse; although some viruses share attachment factors, the mechanisms by which they are internalized differ from virus to virus. Compared to enveloped viruses invading via fusion, nonenveloped viruses enter cells via distinct and complicated processes [1].

Viruses that have entered cells begin to replicate and trigger a series of immune responses, ranging from an immediate innate immune system response to an adaptive immune system response [6]. Innate immune cells recognize and eliminate viruses [7]. Antigen-presenting cells engulf viral antigens and inform the lymph nodes of the existence of viruses in the cell [8]. Cytokines produced in the lymph nodes induce a variety of T helper (T_H_) cell responses [9,10,11]. Viral infections turn on not only innate immunity but also adaptive immunity by helping T_H_ cells secrete antibodies from B cells [12]. Until now, many studies have been conducted on the signal transduction caused by the presence of intracellular viruses; however, little research has been conducted on the induced signaling when viruses interact with cell surface molecules.

The phenomenon of viruses binding to receptors is still an area of ongoing research. Signaling pathways triggered by virions binding to receptors need to be studied. Additionally, their effects on autophagy are complex and poorly understood. A link should be made between the binding of virions to surface molecules and triggered intracellular signaling pathways. Therefore, this review provides an overview of how virus–host interactions affect autophagy. The recent findings on autophagy regulated by ligands will be explained. Future directions of autophagy research in virus–host interactions will be discussed. Understanding these relationships will shed light on viral research.

## 2. Autophagy

### 2.1. Overview of Autophagy

**Figure 1:**
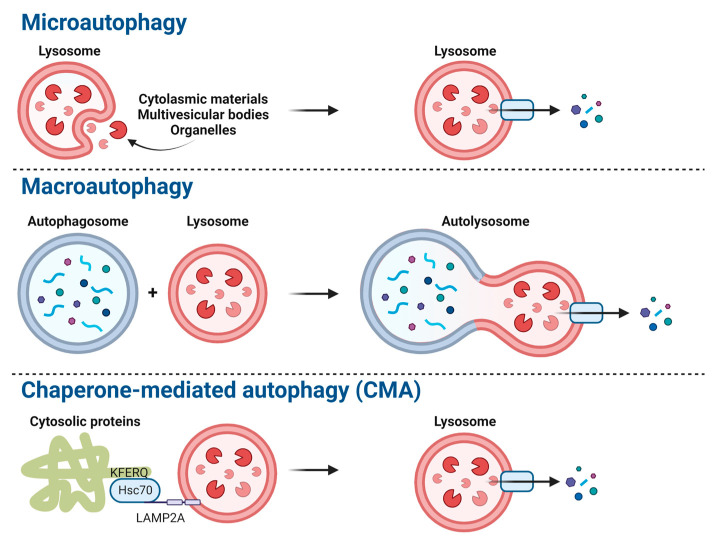
The major autophagic pathways include microautophagy, macroautophagy, and chaperone-mediated autophagy (CMA). This figure was created with biorender.com and accessed on 26 November 2022.

Autophagy is a conserved degradation process for maintaining homeostasis [13]. Autophagy degrades cellular components, including long-lived proteins and damaged organelles in lysosomes. Under starvation, autophagy replenishes building blocks in case molecules are needed urgently [14]. In addition, the autophagic pathway is a defense mechanism against harmful substances, including viruses [15]. Eukaryotic cells degrade undesirable components via microautophagy, macroautophagy, and chaperone-mediated autophagy (CMA) [14,16] (Figure 1). Microautophagy is a non-selective degradation process with respect to lysosomes that directly engulfs intracellular components [17]. CMA degrades proteins that are selectively dependent on lysosomal-associated membrane protein 2A (LAMP2A) with the KFERQ sequence [18]. Macroautophagy utilizes autophagosomes to eradicate substances selectively and non-selectively [19]. Macroautophagy is best-characterized and referred to hereafter as autophagy.

### 2.2. The Mechanism of Autophagy Initiation

**Figure 2:**
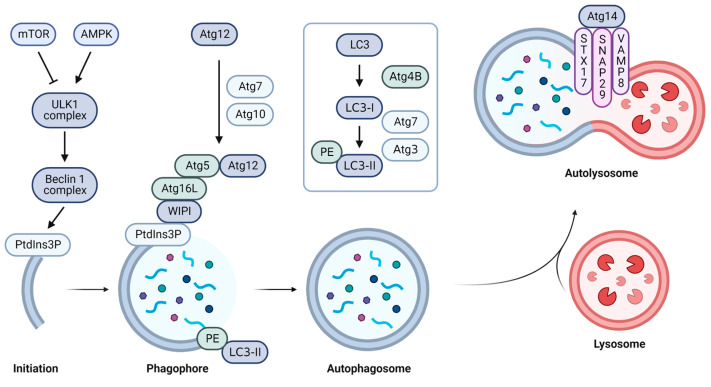
Overview of the canonical autophagy pathway. Canonical autophagy is initiated by signaling according to low intracellular energy. The phagophore is elongated by using the ubiquitin-like conjugation system. The elongated phagophores form autophagosomes which fuse with lysosomes. This figure was created with biorender.com and accessed on 17 January 2023.

The mammalian target of rapamycin (mTOR) is a serine/threonine kinase that senses nutrient levels and activates cell growth and proliferation [20] (Figure 2). mTOR inhibits autophagy by phosphorylating ULK1 and ATG13 [21]. Under starvation, mTOR is inactivated, resulting in the formation of a ULK1 complex [22]. Another nutrient sensor, AMP-activated kinase (AMPK), phosphorylates different sites on mTOR and ULK1 in addition to regulating autophagy [21]. Activated ULK1 complexes (ULK1, FIP200, Atg13, and Atg101) phosphorylate Beclin1 and activate the formation of the Beclin1 complex (Beclin1, VPS34, VPS15, and Atg14) [20,22,23]. VPS34 is a lipid kinase that phosphorylates the three hydroxyl groups of phosphatidylinositol (PtdIns) and changes into phosphatidylinositol 3-phosphate (PtdIns3P) [23,24,25]. Enriched PtdIns3P by VPS34 [26] is the starting point of phagophore formation. PtdIns3P binds to the WD repeat domain phosphoinositide-interacting protein (WIPI) [25]. Atg12 is a ubiquitin-like protein that is conjugated and activated by Atg7 (E1 ubiquitin-activating enzyme) and Atg10 (E2 ubiquitin-conjugating enzyme) [27]. Activated Atg12 is conjugated to Atg5, and the complex binds to ATG16L [28]. The Atg12–Atg5–Atg16L complex is recruited to WIPI, which is bound to PtdIn3P. The recruitment enhances the elongation of phagophores. Meanwhile, light chain 3 (LC3) is cleaved into LC3-I by the Atg4B protease [29]. E1 Atg7 and E2 Atg3 combine LC3-I with phosphatidylethanolamine (PE) to form LC3-II [30]. The lapidated LC3-II binds to the phagophore and is recruited for the membranes to form autophagosomes [31]. After forming the autophagosome, Atg14 binds to the SNARE complex (sytaxin17, SNAP29, and VAMP8) and induces autophagosome–lysosome fusion [32,33].

## 3. Viruses Interacting with Cell Surface Receptors, Triggering Autophagy

Viruses use strategies to enter cells, bind to receptors on the cell surface, and undergo endocytosis [34]. Thus, the binding between a virus and its receptors is considered the first gateway for infecting cells. During the viral infection of cells, the autophagic pathway is regulated by the interaction between a virus and its receptor. It should be noted that the receptors mentioned here are viral receptors on the cell surface and not autophagosome receptors such as p62/SQSTM1.

### 3.1. Inducing Autophagy via the CD46-Cyt-1/GOPC Pathway

CD46 is a protein ubiquitously expressed in various human tissues [35]. CD46 binds to C3b/C4b in order to regulate the complement system of innate immunity [36,37]. CD46 expresses four isoforms in most tissues. The gene expressing CD46 is located at chromosome 1 q3.2 and forms four isoforms via alternative splicing [38]. The extracellular domain contains four short consensus repeats (SCR domain) and alternatively spliced serine/threonine/proline regions (STP domain). Depending on the alternative splicing, the number of STP domains (1 to 3) is determined. The rest of CD46 has a transmembrane domain and a cytoplasmic tail. The isoforms of the cytoplasmic tail are generated from exon 13 and 13/14 by alternative splicing. CD46 has either one of them as an intracellular tail [36].

CD46 is called a pathogen’s magnet [39]. CD46 is the receptor of the measles virus [40], adenovirus [41,42,43,44], herpesvirus 6 [45,46], cytomegalovirus [47], bovine viral diarrhea virus [48], atypical porcine pestivirus [49], and bacterial group A Streptococcus [50]. Various pathogens aberrantly bind to and internalize multiple domains of CD46 (Table 1). Of the pathogens not listed, the binding sites are not mapped yet.

The attenuated measles virus binds to CD46 and is internalized into cells, but the pathogenic measles virus binds to CD150 rather than CD46. The binding of the attenuated measles virus to CD46 induces autophagy. However, autophagy is not activated when the pathogenic measles virus binds to CD150. The binding of the attenuated measles virus to CD46 activates the CD46-Cyt-1/GOPC (Golgi-associated PDZ and containing a coiled-coil motif) pathway [39]. GOPC is a scaffold protein with a PDZ and a coiled-coil domain (CC). It interacts with Cyt-1 via the PDZ domain. GOPC then binds to Beclin1 through the CC domain and interacts with Beclin1-VPS34. This interaction initiates the formation of autophagic vesicles and induces autophagy [51].

CD46 initiates autophagy upon association with the measles virus as well as group A *Streptococcus*. This suggests that CD46 has the potential to induce autophagy when combined with other pathogens; therefore, other viruses with unidentified binding with respect to CD46 are highly likely to cause autophagy. However, what is interesting is that when a pathogen infects, autophagy is initiated to eliminate the pathogen. During virus replication, autophagy is activated to complete the replication of viruses by maintaining the state of the cell [52]. From the perspective of pathogens, it would be interesting to see how autophagy affects infections.

**Table 1:** Pathogens binding to CD46.

Binding Domain in CD46	Ligands	Reference
Short consensus repeats 1 (SCR1)	Measles virus Adenovirus	[53,54,55]
Short consensus repeats 2 (SCR2)	Measles virus	[53,56]
	Adenovirus	[55]
	Human herpesvirus 6	[57]
Short consensus repeats 3 (SCR3)	Human herpesvirus 6	[57,58]

### 3.2. Toll-Like Receptors (TLRs) Launch Autophagy in MyD88-Dependent Manner

#### 3.2.1. TLRs Recognize the Components of Viruses

Toll-like receptors (TLRs) are transmembrane proteins and are pattern recognition receptors (PRRs) [59,60]. PRRs recognize the pathogen-associated molecular patterns (PAMPs) of pathogens as well as damage-associated molecular patterns (DAMPs) when tissue is damaged [61]. Various viruses carry PAMPs and bind to TLRs that activate inflammatory and immune responses [62].

TLRs consist of an extracellular domain, a transmembrane α-helix domain, and a cytoplasmic signaling domain [63]. The extracellular domain consists of a leucine-rich repeat (LRR) motif consisting of 20–30 amino acids. The feature of an LRR is a motif with an LxxLxLxxNxL sequence [63]. This extracellular domain recognizes PAMPs and DAMPs [64]. The transmembrane α-helix domain consists of a single transmembrane helix. The cytoplasmic signaling domain includes a toll/interleukin 1 receptor (IL-1R) homology (TIR) domain. The intracellular signal transduction from TLR occurs in the TIR domain [65].

TLRs are expressed in immune cells, such as dendrite cells, macrophages, neutrophils, and lymphocytes, as well as non-immune cells, such as fibroblast cells or epithelial cells [66]. TLRs are located on the cell surface and in the membranes of subcellular organelles inside cells. TLR3, TLR7, TLR8, TLR9, TLR11, TLR12, and TLR13 are located intracellularly [66]. When intracellular TLRs recognize viral PAMPs (e.g., viral dsRNA, viral ssRNA), they activate signaling pathways for inducing immune responses within cells. It has been well-studied that TLR proteins located in the endosome directly or indirectly recognize PAMPs. The PAMPs bound by the TLRs activate the interferon pathway inside the cell and cause interleukin secretions in order to induce an antiviral immune response [67].

TLRs on the cell surface include TLR1, TLR2, TLR4, TLR5, TLR6, and TLR10 [66]. TLRs on the cell surface are transported from the endoplasmic reticulum (ER) to the plasma membrane via the golgi apparatus [60,68]. TLR2, TLR4, and TLR10 can recognize the proteins (e.g., glycoproteins) of the outer shell of the virus [69,70,71]. Cell surface TLRs also initiate interferon- and interleukin-mediated immune responses, resulting in antiviral responses [72]. This review will focus on TLRs localized on cell surfaces.

#### 3.2.2. Viral Interactions with TLRs Lead to the Initiation of Autophagy

TLR2 is the best-characterized receptor, along with TLR4, among TLRs. TLR2 recognizes several pathogens, such as viruses, bacteria, fungi, and parasites. TLR2 functions as a homodimer or heterodimer (TLR2/TLR1 and TLR2/TLR6). Homodimers recognize lipopolysaccharides (LPSs), porins, lipoproteins, lipoteichoic acid, the peptidoglycan of bacteria, glycoproteins, and the hemagglutinin of viruses. TLR2 and TLR1 recognize bacterial triacylated lipopeptides. TLR2 and TLR6 recognize the glycoproteins of viruses, diacylated lipopeptides, and the lipoteichoic acid of bacteria in addition to the zymosan of fungi [73]. It is known that TLR2 homodimers do not transduce signals into cells, although the reason for this is still unclear [74].

Cell surface TLRs are involved in infections with several viruses (Table 2). TLR2 interacts with the envelope fusion protein F of respiratory syncytial viruses (RSVs) [75], measles virus [76], hepatitis C virus [77], and hepatitis B virus [78]. TLR4 interacts with the H protein of measles virus [76], the glycoprotein B/H of cytomegalovirus (CMV) [79], the glycoprotein gH/gL/gB of herpes simplex virus (HSV)-1 [80].

TLR2 and TLR4 induce intracellular signaling in a myeloid differentiation primary response 88 (MyD88)-dependent manner [90]. Myd88-adaptor-like (MAL) is required to bring MyD88 to TLR2 [91]. The TIR-domain-containing adapter-inducing interferon β (TRIF)-related adapter molecule (TRAM), MAL, and TRIF are required to bring MyD88 to TLR4 [92]. The activation of the MyD88-dependent pathway by cell surface TLRs is mediated by nuclear factor κB (NF-κB) and promotes the expression of inflammatory cytokines [93]. Intracellular TLRs induce antiviral action via interferon signaling pathways mediated by TRIF or MyD88 [94].

Cell surface TLRs activate the autophagy process via adaptor proteins [95]. TLR3, 4, and 7, stimulated by viral binding, bind to the adaptor proteins (MyD88 and/or TRIF) [96,97]. This interaction enhances the binding between adaptor proteins and Beclin 1 [97]. The binding regulates the binding affinity between Bcl-2 and Beclin 1, and Bcl-2 is dissociated [98]. Afterwards, Beclin 1 binding to VPS34 and other Atgs initiates autophagy [99]. HSV-1 induces autophagy rapidly after infection via TLR2-MyD88. In addition, MyD88-deficient THP-1 cells fail to induce autophagy despite infections with HSV-1 [90]. CMV infection showed a rapid increase in the production of lapidated LC3-II, a well-known autophagy marker [100].

Further studies are needed to clarify how the interaction between viruses and TLRs causes autophagy. Fundamentally, the function of TLRs is to induce an antiviral response during viral infection. It will be essential to interpret the role of autophagy triggered by TLRs in the early stage of infection.

### 3.3. Integrins Are Possible Viral Receptors Causing Autophagy

Integrin is an adhesion molecule that plays a role in tissue formation and cell migration via cell-to-cell binding. Integrins function as adhesion molecules by binding to fibronectin, laminins, collagens, and various proteins in the extracellular environment. It is also one of the cell surface proteins responsible for communication between cells. Integrins are composed of an α-chain and a β-chain, and they function by forming a heterodimer. Integrin consists of a large extracellular domain, a single transmembrane domain, and a small intracellular domain. Integrin transmits signals from the inside to outside and signals by ligand binding from the outside to inside. Integrin undergoes conformational changes according to the degree of activation [101]. Integrins are bent in the inactive state; in the activated state, the head portion is open and extended. The binding partner modulates the activity of integrins from both sides. Therefore, inside and outside signaling processes are linked with integrins. The affinity with ligands varies according to intracellular signaling. The ligand binding site becomes accessible, and the binding affinity for ligands increases. Integrin binds to a short ligand motif and transmits a signal. αvβ3, αvβ5, αvβ6, α5β1, and αIIbβ3 recognize the RGD sequence, and α2β1 recognizes the DGE sequence.

The spike protein of SARS-CoV-2 has an RGD sequence, and the possibility of binding to the integrin has been reported [102]. Recently, it has been revealed that integrin can be a receptor for SARS-CoV-2. Angiotensin-converting enzyme 2 (ACE2) is the primary receptor, but β1 and β3 integrins can also be co-receptors. T777, S778, T779, and Y785 near the LC3-interacting region (LIR) of β3 integrin are phosphorylated. Proteins with an LIR are involved in regulating autophagosome formation and maturation. Each of the phosphorylated residues cause integrin to bind distinctively to LC3. Comparing the binding affinity with LC3, T777 has the lowest binding affinity, followed by T779. For S778 and Y785, the affinity is similar. The affinity is highest when T779 and Y785 are double-phosphorylated. Therefore, the differential binding of integrin and LC3 according to the phosphorylation of integrin suggests the possibility that integrin plays a large role in forming autophagosomes.

SARS-CoV-2 binding to integrin β1 is crucial for the infection [103,104]. The conformational states of integrin β1 shift depending on the binding affinity; however, the effect of the binding between SARS-CoV-2 and integrin β1 on autophagy is elusive. Other pathogens binding to integrin β1 trigger autophagy. Integrin α5β1 is a receptor of fibronectin, which binds to the FbaA of group A *Streptococcus* (GAS) [105]. The infection of GAS activates the autophagic pathway dependent on α5β1 and not on TLR2 and TLR4. FbaA binding results in autophagy by inactivating mTOR. *Yersinia enterocolitica* on the membrane activates autophagy dependent on β1 [106].

Integrins activate autophagy by interacting with the LIR motif or by suppressing the mTOR pathway. The β3 integrin has an LIR motif, but not all integrins have the motive. Moreover, integrin-binding partners in the intracellular domain can have LIR motifs and mediate the interaction with LC3. Other pathways can be involved, such as the interaction with the Beclin-1 complex. In addition, how integrins inactivate the AKT-mTOR pathway is poorly understood. Unraveling the signal transduction will be the key in virus–autophagy research (Table 3).

### 3.4. Other Receptors Involved in Inducing Autophagy

The viral protein H of peste des petits ruminants virus binding to NECTIN4 induces autophagy via the AKT-mTOR pathway [153]. The gp41 subunit of human immunodeficiency virus-1 (HIV-1) envelope glycoproteins (Env) binds to C-X-C chemokine receptor type 4 (CXCR4), and this interaction triggers the activation of autophagy [154,155]. The interaction between gp120 and the primary receptor of HIV-1, CD4, is not directly involved in the process. The question of how the interplay between gp41 and CXCR4 activates autophagy has not been resolved. Glycoprotein G on the vesicular stomatitis virus (VSV) attaches to receptors, and the interaction begins the process of the virus entering a host cell via endocytosis [156]. In *Drosophila* S2 cells, autophagy is induced by UV-inactivated VSV or VSV-G virus-like particles. The glycoprotein itself is enough to activate autophagy by binding to toll-7 [157].

## 4. Conclusions

What is the function of autophagy regulated by viral binding? This is a fascinating and important question that assists in determining why autophagy is controlled when a virus hijacks the function of a receptor and enters the cell. Intracellular substances are broken down, and cells refill the building blocks during autophagy. The autophagy activated by viral binding induces the preparation process associated with entry and multiplication. In addition, it can be expected that the quality control of old organelles or proteins can be degraded to optimize the condition of cells.

On the other hand, from the perspective of cells, as the virus binds it can transmit a warning signal to the cell. Therefore, as soon as a virion touches the cell, the cell is ready to eliminate the virus via autophagy or autophagy-induced apoptosis. The molecular mechanism of viral binding autophagy is complicated to understand with the current knowledge, since cells respond differently from viruses; there is still a long way to go in understanding the meaning, and more research is needed.

Viruses use various cell surface molecules that are essential for maintaining cell homeostasis. Each ligand induces a different reaction within a cell, such as cell proliferation, cell differentiation, and cell migration; however, how these distinct signaling pathways are activated is still poorly understood. The binding of a virus to a receptor transmits other signals into the cell. Autophagy research, induced by a virus binding to a receptor, has not been sufficiently studied. Establishing the role of autophagy in viral binding will help in understanding why autophagy is induced. Moreover, although receptors are not primarily involved in immune responses, such as integrins, which are involved in the entry of many viruses, further studies are needed with respect to receptors that induce autophagy upon viral binding.

Antiviral drugs are important in inhibiting viral infections and slowing their spread. Antiviral drugs can target each step of a virus’s life cycle. Understanding the mechanisms of viral infections is essential to developing antiviral drugs. Viruses and receptors are useful targets for antiviral drugs, which block binding from occurring outside a cell. To date, seven substances have been approved by the FDA against four types of viruses (RSV, HSV, HIV, and varicella-zoster virus) [158]. During the SARS-CoV-2 epidemic, binding inhibitors for coronaviruses were proposed. Peptides derived from the binding site of the ACE2 receptor were suggested and can prevent SARS-CoV-2 infection [104,159]. It is crucial to develop antiviral drugs using this idea because some viruses share the same receptors. The drugs can alter cell functions by manipulating ligands or receptors without passing through cell membranes. The drugs are not only important for patient treatments but also have a significant impact on various intracellular signal transduction studies, including autophagy caused by viral binding. Understanding viral entry mechanisms and their effects on autophagy will introduce new methods for treating viruses and various diseases.

## Figures and Tables

**Table 2:** Viruses interact with TLRs.

TLR	Ligands	Reference
TLR2	Respiratory syncytial virus	[75]
	Cytomegalovirus	[81,82]
	Herpes simplex virus-1	[83]
	Epstein–Barr virus	[84]
	Measles virus	[76]
	Hepatitis C virus	[77]
	Hepatitis B virus	[78]
	Varicella-zoster virus	[85]
TLR2/TLR6	Respiratory syncytial virus	[75]
	Dengue virus	[86]
TLR4	Measles virus	[76]
	Cytomegalovirus	[79]
	Herpes Simplex virus-1	[80]
	Ebola virus	[87]
	Respiratory syncytial virus	[88]
TLR10	Human immunodeficiency virus	[89]

**Table 3:** Integrins are involved in virus entry.

Integrins	Virus	Reference
β1	Reovirus	[107]
	Human cytomegalovirus	[108,109]
β3	Human cytomegalovirus	[109]
α1β1	Ross River virus	[110]
α2β1	Rotavirus	[101,111]
	Echovirus	[112]
	Kaposi’s sarcoma-associated herpesvirus	[113]
	Echovirus 1	[114,115]
	Cytomegalovirus	[116]
α3β1	Kaposi’s sarcoma-associated herpesvirus	[117]
	Adenovirus	[118]
α4β1	Rotavirus	[111]
	Infectious bursal disease virus	[119]
α5β1	Foot-and-mouth disease virus	[120]
	Epstein–Barr virus	[121]
	Adenovirus	[122]
α6β1	Cytomegalovirus	[116]
α9β1	Kaposi’s sarcoma-associated herpesvirus	[123]
αMβ2	Adenovirus	[124]
αVβ1	Echovirus 22	[125]
	Adenovirus	[126]
	Foot-and-mouth disease virus	[127]
	Human parechovirus 1	[128]
αVβ3	Cytomegalovirus	[108,116]
	Human parechovirus 1	[128]
	Herpes simplex virus	[129]
	Echovirus 9	[130]
	Coxsackievirus A9	[131]
	Andes virus	[132]
	Adenovirus	[133,134]
	Rotavirus	[135]
	Sin Nombre virus	[136]
	Hantaan virus	[137]
	Human immunodeficiency virus 1	[138]
	Foot-and-mouth disease virus	[139]
	Japanese encephalitis virus	[140]
	Kaposi’s sarcoma-associated herpesvirus	[141]
αVβ5	Adenovirus	[133]
	Kaposi’s sarcoma-associated herpesvirus	[142]
	Epstein–Barr virus	[143]
αVβ6	Coxsackievirus A9	[131]
	Epstein–Barr virus	[143,144]
	Herpes simplex virus	[145,146]
	Foot-and-mouth disease virus	[147,148]
αVβ8	Epstein–Barr virus	[143,144]
	Herpes simplex virus	[145,146]
	Foot-and-mouth disease virus	[149]
α6β1	Papillomavirus	[150]
α6β4	Papillomavirus	[150,151]
αXβ2	Rotavirus	[111]
αIIbβ3	Sin Nombre virus	[152]
	Hantaan virus	[152]

## Data Availability

Not applicable.
